# Orbital apex syndrome secondary to apical periodontitis of a tooth: a case report

**DOI:** 10.1186/s12883-022-02890-0

**Published:** 2022-09-19

**Authors:** Wei Xiang, Hongchun Wei, Luyao Xu, Zhigang Liang

**Affiliations:** Department of Neurology, The Affiliated Yantai Yuhuangding Hospital of Qingdao University, Yantai, Shandong China

**Keywords:** Orbital apex syndrome, Stroke, Odontogenic infection, Cranial nerve injury

## Abstract

**Background:**

Orbital apex syndrome (OAS) is a rare disease with a noticeable mortality rate. Although its etiology has been repeatedly assessed, few reports have concentrated on odontogenic infection. We presented a rare case of OAS secondary to apical periodontitis.

**Case presentation:**

A 61-year-old male was admitted to our hospital for a 3-day history of left orbital and head pain, along with diplopia for 1-day. He also had toothache symptoms before his admission. Due to the atypical early symptoms of orbital apex and cranial nerve injury, no timely and effective diagnosis and treatment were initially provided. However, as the disease progressed and complications occurred, we timely adjusted the diagnosis and successfully controlled the infection. During the one-year follow-up, no recurrence of inflammation was observed; nevertheless, the ptosis and ophthalmoplegia persisted.

**Conclusions:**

OAS is a rare, while severe complication of odontogenic infection. This case had various symptoms and nerve injury in the orbital apical area. When disease is atypical in its early stages, treatment is easily overlooked. Early detection and suspicion of orbital apex-related complications should be heightened.

## Background

Orbital apex syndrome (OAS) is a disorder characterized by involvement of a series of cranial nerves II, III, IV, VI, and it was described as a syndrome involving damage of the oculomotor, trochlear, abducens nerves, and ophthalmic branch of the trigeminal nerve in association with optic nerve dysfunction [1, 2]. OAS can be caused by a variety of factors, with traumatic and neoplastic etiologies, while the infection was scarcely reported. OAS is an infrequent malignant disease. Once the infection spreads, its progression is noteworthy. The present study aimed to report a rare case of OAS secondary to apical periodontitis, in order to assist clinicians in identification of early atypical symptoms of the disease.

## Case presentation

A 61-year-old man, with a history of hypertension and type 2 diabetes mellitus who suffered from toothache for a week, was admitted to the ophthalmology department of our hospital.

**Fig. 1:**
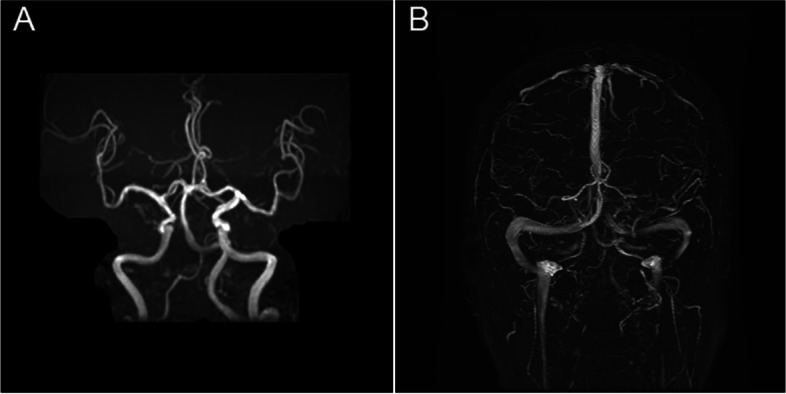
Cerebral MRI findings. **A** MRA shows mild atherosclerotic changes. **B** MRV is normal

**Fig. 2:**
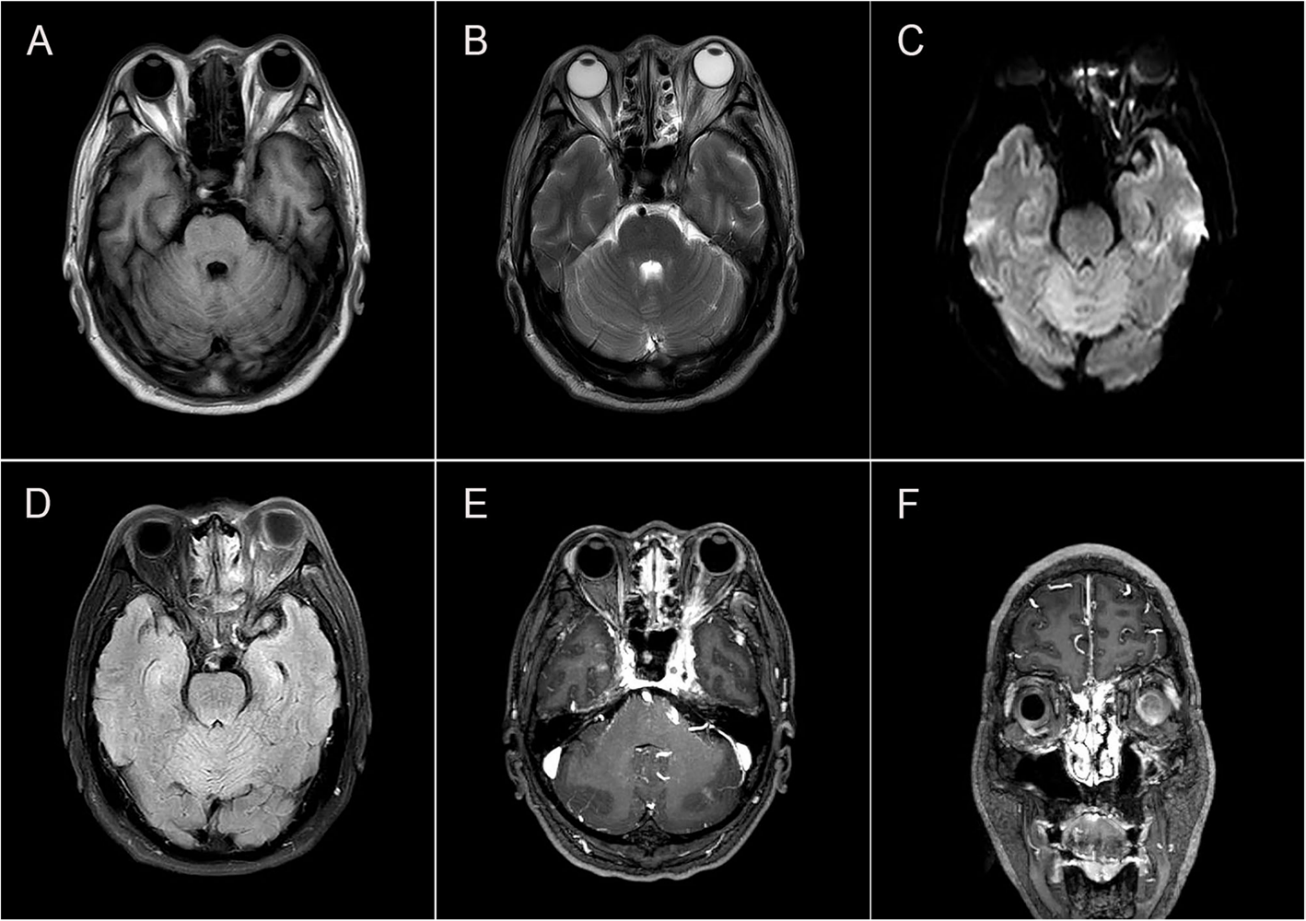
MRI of brain and orbits with gadolinium contrast showing thickened and enhanced medial, lateral, inferior, and supraocular muscles. Both T1- and T2-weighted imaging were hypointense **A**, **B**. DWI and FLAIR sequences were hyperintense **C**, **D** Axial and coronal scans were markedly enhanced **E**, **F**

There was the left orbital pain at three days before his admission, followed by headache and double vision on the left side for one day. Examination showed that the left eye vision was 0.5, and the intraocular pressure (IOP) was 18 mmHg. Besides, proptosis, complete ptosis, conjunctival hyperemia, and nearly frozen ocular motility in all directions were observed. The pupil diameter was around 4.0 mm, and both direct and indirect responses to light were weak. Laboratory tests revealed a leukocyte count of 15.31× 10^^9^/L with a neutrophil preponderance, and a high C-reactive protein level of 35.99 mg/L was measured. The proportion of glycosylated hemoglobin was 12.9%. The computed tomography of the orbit without contrast showed a depression in the right intraorbital wall, which could be related to old fracture, bilateral septal sinus, and maxillary sinusitis. The contrast-enhanced magnetic resonance imaging (MRI) of brain revealed slightly scattered demyelinating changes, which were not pathologically significant. No significant abnormality was observed in magnetic resonance angiography (MRA) and magnetic resonance venography (MRV) (Fig. 1). Following admission, the patient was diagnosed with diabetic ophthalmoplegia and treated with local analgesics, nutritional therapy, and glycemic management. As the patient had toothache one week before admission and his symptoms had not been fully disappeared, after dental examination, he was diagnosed with apical periodontitis and given ceftriaxone sodium (2 g intravenously daily) combined with metronidazole (0.6 g orally three times a day) for anti-inflammatory treatment. However, four days later, the patient had vision loss. Subsequently, on the fifth day of hospitalization, he experienced difficulty in moving his right hand when he got up. Physical examination indicated total ptosis of the left eye and paresis of extra-ocular eye movements. The left pupil diameter was 5 mm, and light reflection disappeared, accompanied by hypoesthesia of the left forehead. The right upper limb’s muscle strength was grade 4, and the right finger nose test was inaccurate, with no abnormality in the other neurological examinations. The National Institutes of Health Stroke Scale (NIHSS) score was 3 points. The MRI of brain showed infarction in the left frontal-parietal lobe and temporal-occipital junction area. Due to the onset of the disease within 4.5 h and the diffusion-weighted MRI (DWI)-fluid-attenuated inversion recovery (FLAIR) mismatch, the patient received intravenous thrombolysis with recombinant tissue plasminogen activator (r-tPA). After intravenous thrombolysis, the patient’s symptoms were improved and the NIHSS score was 2 points. Afterwards, digital subtraction angiography (DSA) was performed and revealed occlusion of the left internal carotid artery, in which the right internal carotid artery was partially compensated by the opening of the anterior communicating artery; and the posterior circulation was partially compensated by the opening of the thin posterior communicating artery. The patient was advised to undergo orbital MRI, which revealed a hyperintense lesion in the medial, lateral, inferior, and supraocular muscles, causing compression of the adjacent optic nerve. The lesion was found to be hypointense on both T1-weighted images (T1WI) and T2-weighted images (T2WI), with significantly contrast enhancement (Fig. 2). Therefore, lumbar puncture was performed that showed cerebrospinal fluid pressure of 200 mmH_2_O, white blood cell count of 11.35 ∗ 10^^6^/L, single-cell nucleus of 79%, glucose level of 4.91 mmol/L, chlorine level of 122.9 mmol/L, and cerebrospinal fluid protein level of 952.5 mg/L. Pseudomonas aeruginosa was found by detection of pathogenic microorganisms using metagenomic next-generation sequencing (mNGS). After a systemic evaluation, the final diagnosis was orbital inflammation caused by apical periodontitis and developed progressively, resulting in multiple neural injuries at the orbital apex. Acute ischemic stroke is caused by occlusion of the internal carotid artery due to a cavernous sinus abscess and inflammation. Therefore, the treatment was changed to linezolid (0.6 g orally every 12 h) and piperacillin-tazobactam (4.5 g intravenously every 8 h), in combination with low-dose steroids (intravenous methylprednisolone, 20 mg for 10 days), antiplatelet aggregation, and reducing intracranial pressure. Simultaneously, dental extraction was performed after dental examination. After medical treatment, the patient’s symptoms were alleviated, accompanying by reduction of pain and swelling, and slightly improvement of vision in the left eye; nonetheless, during the 1-year follow-up, the patient’s visual examination showed only light perception, and the presence of ptosis and ophthalmoplegia was still confirmed.

## Discussion and conclusions

OAS may result from various etiologies, including inflammation, infection, neoplasia, trauma, vasculitis, and even iatrogenic diseases [3]. A previous study showed that odontogenic orbital infections are uncommon, accounting for only 2–5% of all orbital cellulitis cases [4]. The infection spreads to the maxillary sinus and other sinuses through the infected dental fossa, then, to the orbit and surrounding areas, involving the extraocular muscles and optic nerve, and possibly to the cavernous sinus. Even though it can spread via a variety of routes, early orbital involvement following oral infections has been rarely reported [5]. In our case, the uncontrolled diabetes mellitus and recent toothache raised the suspicion of an infectious cause. Because the symptoms of orbital apex nerve injury were manifested slightly later and the pain was not obvious, the inflammation caused by OAS was not promptly identified, and treatment was slightly delayed. However, after the occurrence of acute ischemic stroke, we promptly ruled out contraindications and given intravenous thrombolysis. After accurately investigation of the medical history, the brain and orbital apex examinations were performed based on the patient’s status. Due to the deep location of the cavernous sinus, its complex anatomical structure, and the patient’s age, he was complicated with comorbidity, and performing pathological biopsy was difficult. The absence of pathology biopsy is a major limitation, because it may assist clinicians in confirming the diagnosis and identifying the pathogenesis of diseases. In our study, the patient’s prognosis significantly improved following active and effective anti-infection treatments.

A systematic analysis of odontogenic OAS found that 18 of 21 patients had a history of tooth extraction and three had gingival infections, mainly including invasive fungal and bacterial infections, with a mortality rate of 54% [6]. OAS is a severe and uncommon complication of odontogenic infection with high rates of morbidity and mortality. Early detection of ocular and cranial nerve involvement is critical to the precise diagnosis and treatment. Additionally, a previous study demonstrated that the incidence of OAS was higher in diabetic patients, which could be related to the elevated blood glucose level and immune-mediated nonspecific inflammation [7]. Hyperglycemia is a critical trigger of infection. Therefore, patients with diabetes and OAS should immediately undergo hypoglycemic and steroid treatment to avoid permanent vision impairment. In this case, the patient had a history of diabetes and poor glycemic control. He had numerous lumbar punctures throughout the follow-up period, and the levels of leukocytes and proteins in the cerebrospinal fluid were slightly elevated. However, the patient’s clinical condition was stable, there was no fever or headache, and the inflammatory indices were normal. Thus, we may conclude that the patient’s inflammation may progress from acute infection to chronic inflammation.

In addition to common causes, such as diabetic ophthalmoplegia, the following uncommon disorders were considered during our patient’s assessment. Tolosa-Hunt syndrome was considered first, as it is characterized by periorbital pain and restricted eye movement, while rarely impairing visual acuity [8]. A significant improvement in clinical symptoms following corticosteroid medication is diagnostically advantageous. In the present case, the optic nerve involvement together with the absence of significant pain led to rule out this diagnosis. Furthermore, it is crucial to distinguish other orbital apex illnesses. Previous studies discriminated OAS from superior orbital syndrome and cavernous sinus syndrome according to anatomical characteristics. There were some similarities in terms of etiology, clinical symptoms, and management. Superior orbital fissure syndrome is similar to OAS, while it does not involve the optic nerve, whereas cavernous sinus syndrome includes hypoesthesia of the cheek and lower eyelid in addition to OAS. Supraorbital fissure syndrome can evolve into OAS and cavernous sinus syndrome as the disease progresses [9].

The prognosis of patients with OAS is determined by the etiology, the extent of nerve injury, and the treatment modality. Due to the rarity of odontogenic OAS, there is no large-sample and randomized clinical trial. At present, treatment for odontogenic OAS is primarily empirical based on case reports and case series. Along with broad-spectrum antibiotic therapy, an interdisciplinary collaboration among departments of ophthalmology, stomatology, and neurology is essential [10].

Due to the complexity and density of nerves and blood vessels in the orbital apex area, OAS can be easily misdiagnosed without adequate knowledge and clinical suspicion, resulting in delayed treatment and permanent visual loss or even death. To improve OAS patients’ survival, clinicians should pay further attention to atypical presentation and be aware of possible etiologies in order to provide prompt and effective treatment.

## Data Availability

The data generated or analysed during this study are included in this article.

## Abbreviations

OAS: Orbital apex syndrome
MRI: Magnetic resonance imaging
MRA: Magnetic resonance angiography
MRV: Magnetic resonance venography
NIHSS: National Institutes of Health Stroke Scale
